# Acute mesenteric ischemia diagnosed via computed tomography in a dog following vehicular blunt force trauma: a Case Report

**DOI:** 10.3389/fvets.2025.1562043

**Published:** 2025-04-17

**Authors:** Johnny Altwal, Liz Guieu, Margaret Cook, Jessie Warhoe, Christopher Ray, Kelly Hall

**Affiliations:** Department of Clinical Sciences, Veterinary Teaching Hospital, Colorado State University, Fort Collins, CO, United States

**Keywords:** gastrointestinal trauma, mesenteric ischemia, cranial mesenteric artery, dog, case report

## Abstract

Acute mesenteric ischemia (AMI) refers to a group of vascular disorders that disrupt intestinal blood flow, resulting in intestinal ischemia and necrosis if left untreated. In both humans and dogs, this condition appears to be rare but deadly. There has only been one documented case of traumatic acute mesenteric ischemia in a dog, and the diagnosis was conducted through laparotomy. We present the case of a dog that was struck by a vehicle and subsequently developed traumatic acute mesenteric ischemia, which was diagnosed through computed tomography (CT). The dog presented with profuse hemorrhagic diarrhea, which persisted throughout the hospitalization. The dog’s condition eventually deteriorated as a result of diffuse intestinal ischemia, diagnosed using computed tomography (CT). Due to the anticipated poor prognosis, the dog was euthanized humanely. Traumatic acute mesenteric ischemia in dogs may be an underdiagnosed consequence of vehicular trauma, necessitating further diagnostic investigation in dogs with persistent gastrointestinal signs that are unresponsive to medical therapy.

## Introduction

Acute mesenteric ischemia (AMI) encompasses several vascular disorders that disrupt intestinal blood flow, leading to ischemia and necrosis of the intestines if left untreated (1). Overall, the incidence of AMI in humans is low, occurring in 0.09–0.2% of all acute cases presented in the emergency department (2–4). AMI has been reported secondary to mesenteric arterial or venous thrombosis, as well as marked intestinal vasoconstriction due to hypovolemia (1, 5–7). Several risk factors for AMI in humans have been described. These include atrial fibrillation, heart failure, myocardial infarction, diffuse atherosclerotic disease, abdominal compartment syndrome, vasopressor use, portal hypertension, oral contraceptive use, and trauma (8, 9). Traumatic AMI (tAMI) has been reported in humans following closed, blunt trauma to the abdomen, with abdominal pain typically reported on presentation (8–11). The majority of human tAMI cases underwent surgical interventions that involved intestinal resection and anastomosis, arteriorrhaphy, venorrhaphy, or a combination of these procedures (8–11). In a study involving 13 cases, the mortality rate associated with surgical intervention was 57% (10).

While blunt force traumas are common in dogs (12–14), only one case report of canine tAMI secondary to blunt force trauma has been identified (15). In this case, despite initial supportive care, the dog remained lethargic and anorexic post-discharge and was re-presented to a veterinarian 36 h after the trauma. Abdominal pain prompted abdominal radiographs, which revealed diffusely, markedly distended, gas-filled loops of the intestine (15). Due to suspected mesenteric torsion, the dog underwent exploratory laparotomy, during which necrosis of the cecum and the proximal two-thirds of the colon was identified, along with the absence of palpable pulses in the associated mesenteric arteries (15). Following resection and ileocolic anastomosis, the dog recovered well and was discharged 2 days postoperatively (15).

The present report describes the clinical progression of a dog that, after experiencing blunt trauma, showed persistent gastrointestinal signs despite medical interventions and was ultimately diagnosed with tAMI via computed tomography (CT).

## Case presentation

An 11-year-old male castrated mixed-breed dog (weighing 27.8 kg) was presented to the Colorado State University Veterinary Teaching Hospital Emergency Room after being hit by a car (day 1). The dog was not on any medications at the time and had no pertinent previous medical history.

## Investigation and treatments

The initial assessment revealed tachycardia at 240 bpm with weak femoral pulses, a respiratory rate of 40 breaths per minute with a patent airway, and a systolic blood pressure of 110 mmHg (measured by Doppler). No external bleeding was noted. The dog was ambulatory with a kyphotic posture but showed no signs of lameness. The electrocardiogram (ECG) showed sinus tachycardia with occasional ventricular premature complexes. Pulse oximetry was 100% in room air. The minimum database revealed a packed cell volume (PCV) of 55% and total protein (TP) of 6.0 g/dL, along with hyperlactatemia of 7.4 mmol/L, which was attributed to hypovolemia (Table 1). Thoracic point-of-care ultrasound (TPOCUS) revealed the absence of the glide sign in the left hemithorax, indicating a pneumothorax, and a few B-lines in the right middle lung field compatible with contusions. Abdominal point-of-care ultrasound (APOCUS) showed no evidence of free abdominal fluid (abdominal fluid score (AFS) = 0).

**Table 1:** Serial venous blood gas values recorded throughout the dog’s hospitalization, presented in chronological order.

	Day 1; 10PM^a^	Day 2; 5AM	Day 2; 6PM	Day 3; 5AM	Day 3; 6PM	Day 4; 5AM	Day 4; 7AM	Canine RI	Unit
Hgb:	19.1 (H)	11.9 (L)	11.9 (L)	11.9 (L)	14.1	13.4	9.3 (L)	13–19	g/dL
P_b_	636	638	635	635	630	633	634	600–800	mmHg
pH	Not available^	7.298 (L)	7.407	7.36	7.367	7.306 (L)	7.298 (L)	7.33–7.45	-
PCO_2_	27.6	43.9 (H)	25.5	27.2	21.1 (L)	22.6 (L)	22.8 (L)	24–39	mmHg
PO_2_^*^	52.7 (L)	52.8 (L)	40.9	36 (L)	39.6 (L)	35.9 (L)	85	67–92	mmHg
HCO_3_^−^	Not available^	20.9	15.8	15	11.8 (L)	10.9 (L)	10.8 (L)	15–24	mEq/L
ABE	Not available^	−4.9 (L)	−7.2 (L)	−8.8 (L)	−11.4 (L)	−13.6 (L)	−14.1 (L)	−4 to 4	mmol/L
Na^+^	147	148	147	149	151	154 (H)	156 (H)	142–153	mEq/L
K+	3.5	3.8	3.4 (L)	3.3 (L)	3.8	4.3	3.4 (L)	3.5–5.2	mEq/L
Cl^−^	119	118	124 (H)	126 (H)	134 (H)	134 (H)	131 (H)	110–122	mEq/L
iCa^2+^	1.24 (L)	1.29	1.26	1.30	1.26	1.28	0.95 (L)	1.25–1.5	mmol/L
Glucose	178 (H)	119	113	114	127	123	136	70–141	mg/dL
Lactate	7.4 (H)	1.8	0.9	0.5	1.2	4.9 (H)	3.9 (H)	0.6–3	mmol/L
Creatinine	Not available^	Not available^	0.59	0.63	0.68	1.69	1.78	0.4–1.9	mg/dL
Anion Gap	Not available^	13.5	10.9 (L)	11.8 (L)	9.2 (L)	13.4	16.8	13–24	mEq/L

ABE: absolute base excess, Cl^−^: chloride, HCO_3_^−^: bicarbonate, Hgb: hemoglobin, iCa^2+^: ionized calcium, K^+^: potassium, Na^+^: sodium, PB: barometric pressure, PCO_2_: partial pressure of carbon dioxide, PO_2_: partial pressure of oxygen, RI: reference interval, (H): above the RI, (L): below the RI, −: not applicable. ^a^Time of presentation to Colorado State University’s Veterinary Teaching Hospital Urgent Care. *known inaccuracy of PO_2_ measurement from venous blood gas analysis. ^Analyzer quality control error, so no measurements were available at that time.

A complete blood count (CBC) and chemistry panel (CHEM) were evaluated (Table 2). An increase in creatinine kinase levels was attributed to muscle injuries, while the increases in ALP, ALT, and AST were likely due to a combination of muscle injury, direct trauma to the liver, and liver hypoxia secondary to hypovolemia.

**Table 2:** Blood analyses for the described dog throughout its hospitalization, presented in chronological order.

Complete blood counts				
	Day 1; 10PM^a^	Day 4; 6 AM	Canine RI	Unit
Hgb	19.2	13.6	13–20	g/dL
HCT	54	39 (L)	40–55	%
RBC	7.87	5.63	5.5–8.5	10^6^/μL
MCV	68	69	62–74	fL
MCHC	36	35	33–36	g/dL
ABS RETIC	104.3 (H)	91.4	0–100	10^3^/μL
Platelet count	228	64 (L)	200–500	10^3^/μL
Platelet clumps	Exist	Exist	–	–
WBC	4.7	4.5	4.5–15	10^3^/μL
Segmented neutrophils	3.4	3.4^b^	2.6–11	10^3^/μL
Lymphocytes	0.9 (L)	0.4 (L)	1–4.8	10^3^/μL
Monocytes	0 (L)	0.1 (L)	0.2–1	10^3^/μL
Eosinophils	0.4	Not available	0.1–1.2	10^3^/μL

Chemistry panels				
	Day 1; 10PM^a^	Day 4; 6 AM	Canine RI	Unit
Glucose	174(H)	125 (H)	70–115	mg/dL
Blood urea nitrogen	27	42 (H)	7–30	mg/dL
Creatinine	1.25	1.88 (H)	0.6–1.6	mg/dL
Phosphorus	2.7	2.4 (L)	2.5–6	mg/dL
Total calcium	9.6	8.8 (L)	9–11.5	mg/dL
Magnesium	2.6 (H)	1.9	1.8–2.4	mg/dL
Total protein	5.6	3.7 (L)	5–7	g/dL
Albumin	3.5	2.3 (L)	3–4.3	g/dL
Globulin	2.1	1.4 (L)	1.5–3.2	g/dL
Cholesterol	185	131	130–300	mg/dL
Creatinine kinase	992 (H)	8,966 (H)	50–275	IU/L
Total bilirubin	0.1	9 (H)	0–0.2	mg/dL
Alkaline phosphatase	248 (H)	329 (H)	15–140	IU/L
Alkaline transaminase	1,436 (H)	572 (H)	10–90	IU/L
Aspartate transferase	745 (H)	263 (H)	15–45	IU/L
GGT	0	0	0–9	IU/L
Iron	210	52 (L)	80–270	μg/dL
Sodium	147	155 (H)	142–152	mEq/L
Potassium	4.08	4.18	3.9–5.4	mEq/L
Chloride	112.6	130.9 (H)	108–118	mEq/L

ABS RETIC: absolute reticulocyte count, GGT: gamma-glutamyl transferase, HCT: hematocrit, HGB: hemoglobin, MCHC: mean corpuscular hemoglobin concentration, MCV: mean corpuscular volume, RBCs: red blood cells, RI: reference interval, WBCs: white blood cells, (H): above the RI, (L): below the RI, −: not applicable. ^a^Time of presentation to Colorado State University’s Veterinary Teaching Hospital. ^b^Marked toxic changes to neutrophils noted on the pathology review of the complete blood count, dated 5/30/2024.

An isotonic crystalloid bolus (lactated Ringer’s solution, 25 mL/kg) was administered to treat hypovolemia. Fentanyl and lidocaine constant rate infusions (CRIs) were sequentially administered until trauma-related pain was controlled. During the initial stabilization, the dog vomited once and began passing large volumes of dark, hemorrhagic diarrhea containing pieces of tissue that resembled sloughed intestinal mucosa. At this point, the differential diagnosis included intestinal ischemia and sloughing resulting from hypovolemic shock, gastrointestinal (GI) bleeding associated with coagulopathy, or acute hemorrhagic diarrhea syndrome.

Thoracic radiographs revealed a bilateral pneumothorax (the right side was more affected than the left), a diffuse interstitial pattern, fractures of the right 8th, 9th, and 12th ribs, right-sided subcutaneous emphysema, and mild pneumomediastinum. During image acquisition, the dog experienced a notable episode of hemorrhagic diarrhea. Ampicillin/sulbactam was initiated due to concerns about GI translocation secondary to severe sloughing of the gastrointestinal lining.

The dog was admitted to the critical care unit and fitted with a jugular catheter (MILA triple lumen jugular catheter, 5Fr × 13 cm) and a urinary catheter (Cook Foley urinary catheter, 8Fr). A recheck of the PCV and TP 3 h post-admission indicated a decrease in both PCV (47%) and TP (5.0 g/dL). Repeat APOCUS revealed scant free fluid accumulation near the left kidney and the spleen (AFS = 2/4), which was too minimal for sampling.

Due to recurring severe tachycardia of 200 bpm 4 h post-admission, a unit of fresh frozen plasma was administered over 15 min, which reduced the heart rate (HR) to 120 bpm. Repeat TPOCUS (5 h post-admission) revealed bilateral pneumothorax, with the left side being worse than the right due to a larger region showing the loss of the glide sign on the left. TPOCUS also showed static B-lines in the right middle lung field and revealed no effusion. Although the dog remained asymptomatic for the pneumothorax, a decision was made to perform a left-sided thoracocentesis procedure to alleviate respiratory effort and breathing discomfort caused by concurrent rib fractures. The procedure successfully removed 2.2 L of air from the chest.

Throughout Day 2 of hospitalization, the dog maintained cardiovascular and respiratory stability, with occasional ventricular premature complexes observed during continuous ECG monitoring. Hemorrhagic diarrhea continued throughout the day, and the dog remained anorexic. The dog was continued on intravenous (IV) isotonic crystalloid fluid (lactated Ringer’s solution, with the rate adjusted based on the clinical evaluation of dehydration, estimated ongoing losses, and body weight), IV fentanyl CRI, and IV lidocaine CRI. Potassium chloride (KCl) CRI (IV), maropitant, ondansetron, and pantoprazole were initiated. The venous blood gas (VBG) results revealed normalized lactate levels, with progressive hyperchloremic metabolic acidosis attributed to the diarrhea (Table 1). Both APOCUS and TPOCUS, performed twice that day, did not show any changes compared to Day 1.

On Day 3 of hospitalization, the dog remained hemodynamically stable but showed signs of nausea and had two episodes of regurgitation. The VBG results indicated worsening hyperchloremic metabolic acidosis. APOCUS revealed that the stomach was mildly fluid-distended; however, no free fluid was noted. The dog continued to receive IV fluids adjusted to his needs, along with analgesics (fentanyl and lidocaine), KCl CRI, maropitant, ondansetron, and pantoprazole. Metoclopramide CRI was initiated due to concerns about the risk of functional ileus. The urinary catheter was removed, and continuous ECG monitoring was discontinued. A nasogastric tube was placed at the end of Day 3, and the stomach was emptied (450 mL of fluid aspirated) with plans to initiate feeding the following morning. Fentanyl and lidocaine were discontinued on the evening of Day 3 due to ensure the comfort of the dog.

Overnight, from Day 3 to Day 4, the dog’s condition worsened, with more frequent episodes of hemorrhagic diarrhea. Around 5 a.m., there was an acute deterioration in the dog’s health, characterized by dull mentation, sinus tachycardia (200–240 bpm), thready femoral pulses, a Doppler blood pressure of 90 mmHg, and a fever of 104.2°F. Abdominal palpation did not reveal any discomfort. A repeat VBG test revealed hyperlactatemia (4.9 mmol/L), a mixed acid–base disorder, and an increased level of creatinine. Cardiac POCUS confirmed the diagnosis of hypovolemia, while APOCUS did not show any abdominal effusion; however, intestinal loops were diffusely dilated. TPOCUS showed the continued presence of a pneumothorax with no glide sign observed bilaterally. Fluid resuscitation was initiated with isotonic crystalloids (lactated Ringer’s solution, 20 mL/kg) and increased to 2 units of fresh frozen plasma and 1 unit of packed red blood cells. Fentanyl and lidocaine CRI were restarted. Despite undergoing therapy, cardiovascular stability was not regained. The CBC and CHEM findings confirmed the suspicion of sepsis (Table 2). A coagulation panel indicated disseminated intravascular coagulopathy (DIC) (Table 3). Concerns regarding sepsis led to an increase in antibiotic therapy (which included ampicillin/sulbactam and enrofloxacin). A second thoracocentesis procedure was performed, during which 1.4 L of air was removed from the left thorax.

**Table 3:** Coagulation profile of the described dog on Day 4 of hospitalization.

	Day 4; 6 AM	Canine RI	Unit
Prothrombin time	12 (H)	7.4–9.4	Seconds
Activated partial thromboplastin time	18.5 (H)	9.8–13.3	Seconds
D-dimer concentration	1.05 (H)	0.03–0.4	μg/dL
Quantitative fibrinogen	385 (H)	123–210	mg/mL
Antithrombin III	78 (L)	104–162	%
Fibrin degradation products	>5 and <20 (H)	0–4	

**Figure 1:**
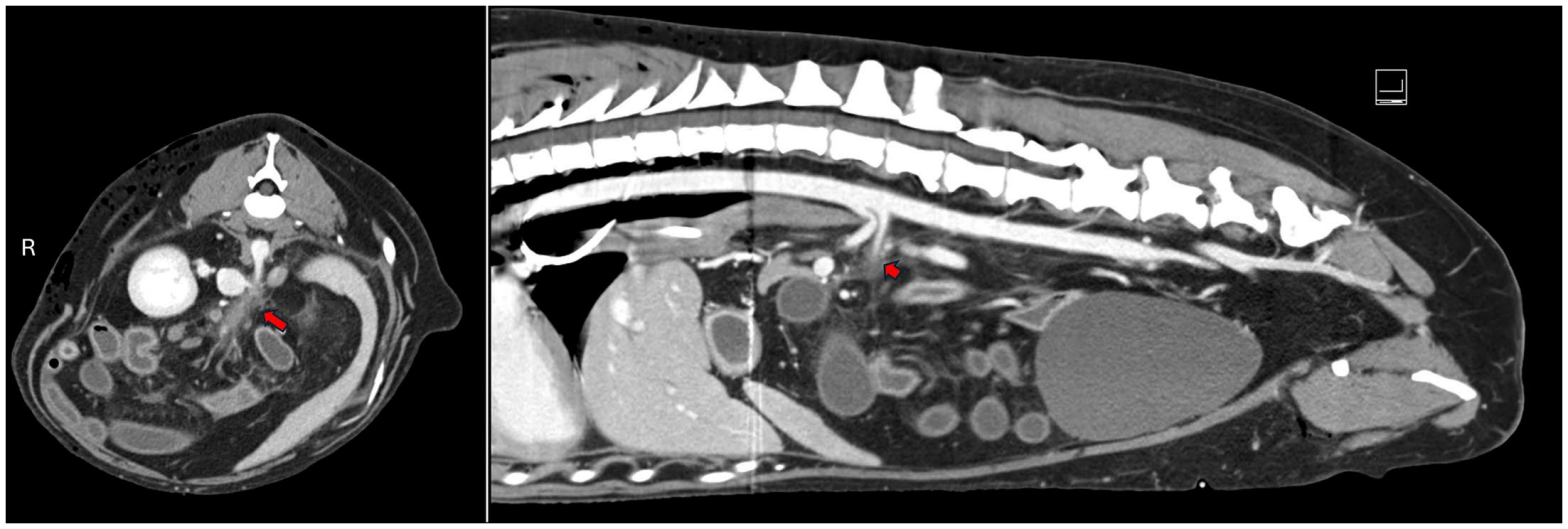
Transverse and sagittal abdominal CT images showing a thrombus in the cranial mesenteric artery, with an associated filling defect (marked by the red arrow). AMI: acute mesenteric ischemia; tAMI: traumatic acute mesenteric ischemia; CMA: cranial mesenteric artery.

An emergent CT scan with contrast was performed, revealing several findings: a thrombus in the cranial mesenteric artery (CMA) with secondary ischemia of the jejunum, ileum, and colon; a bilateral pneumothorax and pneumomediastinum; focal soft tissue attenuating pulmonary regions with internal gas, indicative of either contusions and/or pneumatocele; a small volume of peritoneal effusion; right rib fractures (8, 9, 12); moderate subcutaneous emphysema on the right; and small renal infarcts. An abdominal ultrasound confirmed the presence of the thrombus in the CMA, and an attempt at abdominocentesis was unsuccessful (Figure 1). An assessment by the General Surgery department concluded that radical resection of the ischemic segments and anastomosis of the remaining bowel would be necessary. Due to uncertain to unfavorable short-and long-term prognosis, the owners decided to opt for humane euthanasia.

## Discussion

This case report is the second to describe a case of tAMI in veterinary medicine, with a different clinical course than the previously reported case (15). This report includes the documentation of the CMA thrombus through CT angiography and abdominal ultrasound. With only one previously reported case of canine tAMI, this complication remains rare and may be underdiagnosed. Notably, tAMI has only been documented in dogs following blunt force trauma; it remains unclear whether tAMI can occur as a result of penetrating injuries.

In this present report, the dog was found to have a thrombus in his CMA, which is equivalent to the superior mesenteric artery (SMA) in humans. In humans, either an embolus that originates from the heart or a thrombus that develops due to pre-existing atherosclerotic disease is generally responsible for AMI involving the SMA (1). Trauma has also been described as an underlying cause for AMI associated with SMA occlusion, with several cases reporting tAMI following blunt abdominal trauma in humans (8–11). The pathophysiology of tAMI remains largely unknown, but it appears to involve shock and associated prolonged mesenteric vessel vasoconstriction, leading to gut barrier disruption, bacterial translocation, and irreversible bowel necrosis. This is followed by secondary marked systemic inflammation, reperfusion injury, free radical release, DIC, and septicemia (15–18). In one study involving 30 dogs that had experienced severe trauma, one-third were found to be hypercoagulable on TEG, which was the predominant trauma-induced coagulopathy observed in this cohort of dogs (19). Due to the rarity of tAMI reported in veterinary medicine, trauma-induced coagulopathy could be a contributing factor to thrombosis formation. Non-traumatic causes of mesenteric thrombosis and ischemia have been reported rarely in dogs (20, 21).

In human medicine, abdominal pain accompanied by lactic acidosis, without other apparent illnesses, raises suspicion of AMI and prompts CT angiography for the early detection of thrombotic disease (22). From the reported cases of tAMI in humans, some aspects of the clinical presentations are noteworthy, as similar signs could be observed in dogs with tAMI. All human cases had abdominal pain and some degree of cardiopulmonary distress within 6 h of hospitalization (8–11). In four cases, the patients experienced cardiopulmonary arrests perioperatively: one died before the operation, while three others died postoperatively (two after arteriorrhaphy and one after colonic resection and anastomosis) (9–11). Autopsy findings were available for one of these cases, revealing large thrombi in the SMA, extensive necrosis of the intestinal loops and ascending colon, and blood-tinged fluid in the abdominal cavity (9). In another report, the patient presented with respiratory distress and was successfully treated with resection and anastomosis (8). This patient underwent a preoperative contrast CT scan that showed free air in the abdomen, a filling defect at the origin of the SMA with an abrupt cessation of contrast enhancement, and no contrast enhancement in the jejunum (8).

It is noteworthy to compare the clinical presentation and progression of the two dogs presented in the previous (15) and present case reports. The dog in the previous report was in the back seat of a car that was struck from behind but did not require immediate medical attention (15). The following day, the owners sought veterinary care due to anorexia and tremors in the pelvic limbs. Back pain was identified upon evaluation, and the dog was discharged from the hospital with supportive care (15). The dog was re-presented to a veterinarian 36 h after the trauma due to persistent lethargy and anorexia. During this visit, abdominal radiographs were taken, which revealed diffusely distended GI loops, raising the concern of mesenteric torsion. The dog was anesthetized for an exploratory laparotomy, during which a necrotic cecum and regions of the colon with no palpable pulses were identified. This necessitated resection and ileocolic anastomosis, successfully treating the condition (15). In contrast, the dog in the present report sustained direct, major blunt force trauma to the abdomen and thorax, requiring immediate medical attention. This dog had a significantly greater extent of injury, as evidenced by severe hypovolemic shock, bilateral pneumothorax, rib fractures, and subcutaneous emphysema at presentation. While the dog in our study did not show overt signs of GI loop distention and abdominal discomfort, it did present with hemorrhagic diarrhea, which worsened over time, along with episodes of regurgitation and signs of nausea noted on Day 3 of hospitalization. The authors propose an increased index of suspicion for tAMI in dogs that have sustained blunt force trauma and present with or develop a combination of the following signs: hemorrhagic diarrhea, nausea/vomiting, and/or significant abdominal pain associated with or without intestinal loop dilation during hospitalization. Other differential diagnoses to consider in similar cases include traumatic pancreatitis, bile peritonitis, and uroabdomen.

In human patients, the imaging modality of choice when AMI is suspected is CT angiography (1, 22). However, this recommendation does not exist in veterinary medicine. In the previously published canine tAMI report, only abdominal radiographs were obtained, which showed diffusely gas-dilated intestines, prompting a primary differential diagnosis of mesenteric torsion (15). It was only after laparotomy, resection of necrotic, pulseless bowel with ileocolic anastomosis, and histopathologic evaluation of the resected bowel that tAMI was diagnosed (15). This is the first report of abdominal blunt trauma in a dog that documented a thrombus in the CMA, accompanied by an arterial filling defect and bowel ischemia using CT angiography. It is unclear whether admission with abdominal radiographs or trauma CT would have been helpful in developing an early suspicion of tAMI. Due to the lack of imaging until Day 4 of hospitalization, when the dog developed recurrent signs of shock, it is unknown when the thrombus formed and whether it would have been detected on CT at the initial presentation. Persistent GI signs following trauma should prompt abdominal imaging. Additional research is needed to evaluate the utility of screening abdominal radiographs, abdominal ultrasound, and CT angiography in detecting tAMI, as well as the best timing for such imaging. In human patients, radiographs are not recommended for the evaluation of intestinal ischemia due to the low sensitivity of this modality in detecting ischemic pathology (1). Abdominal radiographs may only be useful in dogs if there is sufficient gas dilation, divergence from normal bowel appearance, such as in the previous canine tAMI report, or free gas in the abdomen to prompt further intervention. It is important to mention that access to veterinary CT angiography is both limited and potentially prohibitively expensive for pet owners.

The role of trauma-induced or associated coagulopathy in dogs with suspected tAMI should also be considered. In humans, it has been established that trauma can acutely induce a hypocoagulable state with hyperfibrinolysis, which can later transition to a hypercoagulable state, promoting thrombotic phenotypes (23). These thrombotic phenotypes can lead to thromboembolisms and multiple organ failure (23). The coagulation profile in this dog indicated DIC on day 4, but his coagulation profile at presentation and during the first 3 days of hospitalization is unknown. However, in severely injured trauma cases, a coagulation evaluation should be considered to detect and address hemostatic abnormalities. Point-of-care coagulation analyzers, such as TEG or the viscoelastic coagulation monitor (VCM Vet®, Entegrion, Durham, North Carolina 27,703), may be useful in blunt trauma cases like the one described. TEG-guided resuscitation of trauma patients has been reported to improve outcomes (24). In a previous prospective study, TEG was shown to be useful in distinguishing between hypercoagulable and hypocoagulable states among dogs with DIC (25). Notably, the use of the VCM for hypercoagulable state detection has been demonstrated to have poor overall performance (26). The benefit of detecting early hypercoagulable states in trauma patients is the ability to initiate prophylactic anticoagulant therapy with medications such as heparin, enoxaparin, or apixaban in a timely manner. In human patients diagnosed with AMI, anticoagulation with heparin is recommended upon diagnosis (1). It is reasonable to consider that a similar recommendation could be implemented in veterinary patients with a hypercoagulable state identified on TEG. Neither TEG nor viscoelastic testing was performed on this patient.

For humans, the recommended treatment for AMI is generally open laparotomy if there are hemodynamic abnormalities and/or evidence of peritonitis (1). If the patient is hemodynamically stable without signs of peritonitis, endovascular approaches have been documented as a more minimally invasive approach, which tends to result in lower mortality compared to open surgery (1). Reported endovascular approaches include aspiration embolectomy, SMA thrombolysis using a biosynthetic form of human tissue plasminogen activator, antegrade stenting, and retrograde stenting (1). Based on the success of open laparotomy in the previous case report of canine tAMI (15), open laparotomy with resection of the affected intestines and anastomosis of healthy segments could be considered a feasible treatment approach in similar cases. Given that no clinical study has been performed to analyze the effectiveness of open laparotomy in successfully treating canine tAMI, the authors cannot recommend open laparotomy for all cases of tAMI. It is currently unknown whether early initiation of anticoagulant medications in at-risk patients could reduce the need for abdominal surgery in dogs diagnosed with partial tAMI. As access to advanced imaging (CT) becomes more common in veterinary medicine and endovascular options become more widely available, a minimally invasive treatment approach for AMI in dogs may be possible in the future. A final recommendation for the management of humans with AMI is to initiate broad-spectrum antibiotic therapy immediately after the detection of AMI, continuing for at least 4 days due to the high risk of GI bacterial translocation and sepsis (1). A similar approach could be considered for veterinary patients with tAMI.

This is the first case report of tAMI in a dog that sustained blunt vehicular trauma, with the diagnosis made using CT and subsequent ultrasonographic confirmation. A limitation of this report is that a necropsy was not performed. This report aims to re-establish tAMI as a potential consequence of blunt force trauma among veterinarians so that early diagnosis and treatment can be initiated. In veterinary trauma patients with persistent GI signs despite appropriate supportive medical care and absent abdominal effusion on APOCUS, coagulation profile testing and CT angiography should be considered early in the diagnostic workup. With quick recognition of tAMI, administration of anticoagulant medications, and surgical intervention as needed, improved patient outcomes may be achieved (15).

## Data Availability

The original contributions presented in the study are included in the article/supplementary material, further inquiries can be directed to the corresponding author/s.

## References

[1] Bala M Catena F Kashuk J Simone BD Gomes CA Weber D. Acute mesenteric ischemia: updated guidelines of the world Society of Emergency Surgery. World J Emerg Surg. (2022) 17:54. doi: 10.1186/s13017-022-00443-x 36261857 PMC9580452

[2] Stoney RJ Cunningham CG. Acute mesenteric ischemia. Surgery. (1993) 114:489–90. 8367801

[3] Acosta S Björck M. Acute thrombo-embolic occlusion of the superior mesenteric artery: a prospective study in a well defined population. Eur J Vasc Endovasc Surg. (2003) 26:179–83. doi: 10.1053/ejvs.2002.1893, PMID: 12917835

[4] Duran M Pohl E Grabitz K Schelzig H Sagban TA Simon F. The importance of open emergency surgery in the treatment of acute mesenteric ischemia. World J Emerg Surg. (2015) 10:45. doi: 10.1186/s13017-015-0041-6, PMID: 26413147 PMC4583757

[5] Acosta S. Mesenteric ischemia. Curr Opin Crit Care. (2015) 21:171–8. doi: 10.1097/MCC.0000000000000189 25689121

[6] Clair DG Beach JM. Mesenteric Ischemia. N Engl J Med. (2016) 374:959–68. doi: 10.1056/NEJMra1503884 26962730

[7] Brandt LJ Boley SJ. AGA technical review on intestinal ischemia. Gastroenterol. (2000) 118:954–68. doi: 10.1016/S0016-5085(00)70183-1, PMID: 10784596

[8] Khan K Bunajem F Alkhan F. Post traumatic arterial occlusive mesenteric ischemia: a rare case report. Radiol Case Rep. (2021) 17:473–6. doi: 10.1016/j.radcr.2021.11.017 34950276 PMC8671805

[9] Hosseini M Hedjazi A Bahrani M. Missed opportunities for diagnosis of post-traumatic thrombosis: a case series and literature review. J Forensic Sci. (2014) 59:1417–9. doi: 10.1111/1556-4029.12453, PMID: 24593035

[10] Courcy PA Brotman S Oster-Granite ML Soderstrom CA Siegel JH Cowley RA. Superior mesenteric artery and vein injuries from blunt abdominal trauma. J Trauma. (1984) 24:843–5. doi: 10.1097/00005373-198409000-00012, PMID: 6481836

[11] Collaco IA Diorio AC Nasr A da Silva FC Cecilio WAC de Toledo-Filho RC. Mesenteric thrombosis in patient victim of blunt abdominal trauma with fatal outcome. ABCD Arq Bras Cir Dig. (2010) 23:58–60. doi: 10.1590/S0102-67202010000100014

[12] Hall KE Holowaychuk MK Sharp CR Reineke E. Multicenter prospective evaluation of dogs with trauma. J Am Vet Med Assoc. (2014) 244:300–8. doi: 10.2460/javma.244.3.300, PMID: 24432962

[13] Hayes G Mathews K Doig G Kruth S Boston S Nykamp S. The acute patient physiologic and laboratory evaluation (APPLE) score: a severity of illness stratification system for hospitalized dogs. J Vet Intern Med. (2010) 24:1034–47. doi: 10.1111/j.1939-1676.2010.0552.x, PMID: 20629945

[14] Talbot CT Raffe MR Boller M Edwards ML Hall KE. ACVECC-veterinary committee on trauma registry report 2020-2021. J Vet Emerg Crit Care. (2024) 34:517–23. doi: 10.1111/vec.13436, PMID: 39494807 PMC11650945

[15] Hamilton TR Thacher CW Forsee KM Nakamura RK. Trauma-associated acute mesenteric ischemia in a dog. J Vet Emerg Crit Care. (2010) 20:595–600. doi: 10.1111/j.1476-4431.2010.00582.x, PMID: 21166981

[16] Yasuhara H. Acute mesenteric ischemia: the challenge of gastroenterology. Surg Today. (2005) 35:185–95. doi: 10.1007/s00595-004-2924-0, PMID: 15772787

[17] Turnage RH Guice KS Oldham KT. Endotoxemia and remote organ injury following intestinal reperfusion. J Surg Res. (1994) 56:571–8. doi: 10.1006/jsre.1994.1091, PMID: 8015313

[18] Crissinger KD Granger DN. Mucosal injury induced by ischemia and reperfusion in the piglet intestine: influences of age and feeding. Gastroenterol. (1989) 97:920–6. doi: 10.1016/0016-5085(89)91498-4, PMID: 2506102

[19] Abelson AL O'Toole TE Johnston A Respess M de Laforcade AM. Hypoperfusion and acute traumatic coagulopathy in severely traumatized canine patients. J Vet Emerg Crit Care. (2013) 23:395–401. doi: 10.1111/vec.12073, PMID: 23855637

[20] Rudinsky AJ Parker VJ Guillaumin J. Mesenteric thrombus associated with pulmonary, splenic, portal, and caval thrombi in a dog that was presented for an acute abdomen. Can Vet J. (2016) 57:1072–6. PMID: 27708446 PMC5026148

[21] Lerman O Israeli I Weingram T Benzioni-Bar H Milgram J Shipov A. Acute mesenteric ischemia-like syndrome associated with suspected Spirocerca lupi aberrant migration in dogs. J Vet Emerg Crit Care. (2019) 29:668–73. doi: 10.1111/vec.12891, PMID: 31701668

[22] Oldenburg WA Lau LL Rodenberg TJ Edmonds HJ Burger CD. Acute mesenteric ischemia: a clinical review. Arch Intern Med. (2004) 164:1054–62. doi: 10.1001/archinte.164.10.1054 15159262

[23] Moore EE Moore HB Kornblith LZ Neal MD Hoffman M Mutch NJ. Trauma-induced coagulopathy. Nat Rev Dis Primers. (2021) 7:30. doi: 10.1038/s41572-021-00264-3, PMID: 33927200 PMC9107773

[24] Gonzalez E Moore EE Moore HB Chapman MP Chin TL Ghasabyan A. Goal-directed hemostatic resuscitation of trauma-induced coagulopathy: a pragmatic randomized clinical trial comparing a viscoelastic assay to conventional coagulation assays. Ann Surg. (2016) 263:1051–9. doi: 10.1097/SLA.0000000000001608, PMID: 26720428 PMC5432433

[25] Wiinberg B Jensen AL Johansson PI Rozanski E Tranholm M Kristensen AT. Thromboelastographic evaluation of hemostatic function in dogs with disseminated intravascular coagulation. J Vet Intern Med. (2008) 22:357–65. doi: 10.1111/j.1939-1676.2008.0058.x 18346141

[26] Hennink I Peters L van Geest G Adamik KN. Evaluation of a viscoelastic coagulation monitoring system (VCM vet®) and its correlation with Thromboelastometry (ROTEM®) in diseased and healthy dogs. Animals (Basel). (2023) 13:405. doi: 10.3390/ani13030405, PMID: 36766294 PMC9913587

